# Dynamics and Strength of Circular Tube Open Wagons with Aluminum Foam Filled Center Sills

**DOI:** 10.3390/ma14081915

**Published:** 2021-04-12

**Authors:** Oleksij Fomin, Mykola Gorbunov, Alyona Lovska, Juraj Gerlici, Kateryna Kravchenko

**Affiliations:** 1 Department of Cars and Carriage Facilities, State University of Infrastructure and Technologies, Kyrylivska Str., 9, 04071 Kyiv, Ukraine; fomin1985@ukr.net; 2 Department of Railway, Automobile Transport and Handling Machines, Institute of Transport and Logistics, Volodymyr Dahl East Ukrainian National University, Central Avenue 59a, 93400 Sewerodonetsk, Ukraine; gorbunov0255@gmail.com; 3 Department of Wagon Engineering and Product Quality, Ukrainian State University of Railway Transport, Feuerbach Square 7, 61050 Kharkov, Ukraine; alyonaLovskaya.vagons@gmail.com; 4 Department of Transport and Handling Machines, Faculty of Mechanical Engineering, University of Zilina, Univerzitna 1, 010 26 Zilina, Slovakia; juraj.gerlici@fstroj.uniza.sk

**Keywords:** circular tube open wagons, carrying structure, aluminum foam, dynamics, strength

## Abstract

The study deals with an application of aluminum foam as an energy-absorbing material for the carrying structure of a rail car. The material is particularly recommended for circular tube carrying structures. The authors conducted mathematical modeling of dynamic loads on the carrying structure of an open wagon that faces shunting impacts with consideration of the center sill filled with aluminum foam. It was established that the maximum accelerations on the carrying structure of an open wagon were 35.7 m/s^2^, which was 3.5% lower in comparison with those for a circular tube structure without a filler. The results obtained were proved by computer modeling. The strength of the carrying structure of an open wagon was also calculated. It was established that aluminum foam applied as a filler for the center sill decreased the maximum equivalent stresses in the carrying structure of an open wagon by about 5% and displacements by 12% in comparison with those involving the circular tube carrying structure of an open wagon without a filler. The natural frequencies and the oscillation modes of the carrying structure of an open wagon were defined. The designed models of the dynamic loading of the carrying structure of an open wagon were verified with an F-test.

## 1. Introduction

The leading position in the global transportation market can be maintained in the rail industry by introducing new innovative engineering solutions in terms of the design at wagon manufacturing facilities. The carrying structure of a rail car is one of the most loaded assembly units regarding operation and material intensity. Therefore, improvements and optimization solutions for the carrying structure of a rail car require particular attention. This requires not only optimal characteristics of the carrying structure in terms of material intensity, but also the application of non-typical materials that enable us to decrease the tare weight and comply, at the same time, with the required strength characteristics. Besides, the issue of lower operational dynamic loading enables us to reduce the damage in the carrying structure, satisfy the requirements for dynamics and strength within the normative values, extend the life cycle of cars, etc. This can be reached by applying energy-absorbing materials in the carrying structures of rail cars. It should be mentioned that the aluminum foam used as a structural material for a long time in the mechanical engineering all over the world has proved its efficiency [1,2,3,4,5]. Article [6] (p. 11) suggests that it is advantageous to use the studied foam for the development of energy absorber elements to improve vehicle crashworthiness during low-speed impact. Metal foams belong to a particular class of metals with an interesting mix of mechanical and physical properties. Characterized by low density and high strength, metal foams show a stiffness/weight ratio five times greater than the bulk material and a large plateau in the compressive stress-strain diagram [7] (p. 55).

However, the issue of an application of aluminum foam in the structures of rail cars has not been discussed yet. Therefore, substantiating the application of aluminum foam for carrying the structures of rail cars is an urgent task nowadays.

## 2. Analysis of Recent Research and Publications

Every newly designed or significantly modified support part of a railway bogie must meet strict criteria related to its strength and fatigue life. Research [8,9] presents key changes of the structure of an original goods wagon bogie frame, conditions, and results strength analyses of unfavorable load cases, for which values of load forces come out from valid standards.

Tools for computer simulations are widely used in the field of a rail vehicle design. These means of virtual reality allow performing static analysis of rail vehicle parts and dynamic analysis of a rail vehicle multibody system [10,11].

Article [12] presents the structural design of the freight railway wagon with variable use of loading space with regard to the safe operation and assessment of the properties by the calculation methods of simulation analysis. Based on computer-aided simulation analysis, they optimized the chassis frame of the wagon. This wagon will be able to offer even more capacity and utilize fewer resources and energy than current wagons for intermodal transport.

Paper [13] describes the most significant and innovative research and development design solutions and computational procedures for a new structure of a railway tank wagon. A strength assessment of a new structure of railway tank wagon is presented. For validation of the new construction assemblies, they created a substitute simulation model.

Peculiarities in the determination of the strength for the carrying structure of a wagon are studied in [14]. It deals with the reasons for defects in components of the wagon body. The authors suggest installing reinforcing elements in the interaction zone between the center sill and the bolster beam.

The research into the structural peculiarities of BCNHL wagons is given in [15]. The study outlines some possible ways to improve the technical and economic parameters of wagons for higher operational efficiency. But measures to decrease the dynamic loading on the carrying structure of the wagons are not considered. 

The structural and element analysis of a BOXN25 open wagon with the finite element method is conducted in [16]. The study deals with the strength calculation and the modal analysis of the wagon structure. The strength of the carrying structure of a 12-757 open wagon with operational wear-outs typical for a 1.5-life cycle is researched in [17]. The study gives an improved dynamic loading on the open wagon in operation and the strength calculation for the carrying structure.

However, the authors do not suggest any measures to improve the carrying structure of wagons to ensure strength under operational loading modes.

The measures for lower dynamic loading on the carrying structure of an open wagon under train ferry transportation are proposed in [18]. The authors present a device that can offer viscous resistance to displacements of the body and be used for fixation of rail cars on the train ferry deck. Its use enables us to decrease the dynamic loading on the body undersea transportation by 30% in comparison with that at a standard fixation pattern.

However, the study does not touch on the issue of improvements in the carrying structure of a rail car for lower dynamic loads in operation.

Study [19] presents peculiarities to be considered during the optimization of carrying structure components for a rail car with an application of the graph-analytical method. The strength calculation was conducted by the finite element method in LIRA-software (version 9.6, Lira-Soft, Kyiv, Ukraine).

The optimization of open-type freight wagon bodies with the bearing floor is given in [20]. The study presents an algorithm of combined structural and parametric optimization for the sidewall and the frame of an open wagon with the bearing floor intended for the axial load of 25 tons. The authors propose optimization techniques for the metal structure of a wagon body. However, the determination of dynamic loads on the carrying structure of a wagon with the proposed implementations is not considered. 

The structural optimization concept of a wagon body of aluminum sandwich panels is given in [21,22]. The characteristic search function for the optimal combination is determined by the maximum stresses and displacements. However, the study does not present the mechanism of how to decrease the longitudinal dynamic loading on the wagon with consideration of the proposed panels.

Aluminum foams are a new class of materials with promise of an improvement of vehicle crashworthiness, combining the properties derived from the cellular structure, in particular the lightness, with the typical behavior of metals [23]. With comparable mechanical properties, it is two-thirds lighter than steel [1,5]. The use of high-strength aluminum alloys for the load-bearing structures of freight wagon bodies will reduce the tare of the wagon to 50% [24,25]. Also, aluminum alloys have high corrosion resistance [24,25].

Flexural studies on foam-filled thin walled aluminum-extruded sections showed higher resistance to bending (7.5 kN) against empty Al-sections (5.8 kN) [26]. The optimum combination of high sound absorption coefficient and frequency range occurred in a crushed foam with good cell interconnectivity.

Research [27] (p. 247) presented the compression tests of specimen aluminum foams. The curves were characterized by a typical initial elastic response, followed by a deformation plateau with a positive slope and finally a transition to densification. The compression strength (at the begging of the plateau with the positive slope) was 5 MPa and the stress variations in the elastic regime were found to be nearly linear.

## 3. Objective and Tasks of the Article

The objective of the article is to reveal peculiarities of the dynamic loading on the carrying structure of a circular tube open wagon with consideration of aluminum foam as filler for the center sill. To solve this goal, the following tasks are defined: mathematical modeling of dynamic load; determination of the strength of the load-bearing structure; computer simulation of dynamic loading; modal analysis; verification of the developed models of dynamic loading.

## 4. Presentation of the Basic Material of the Study

**Figure 1:**
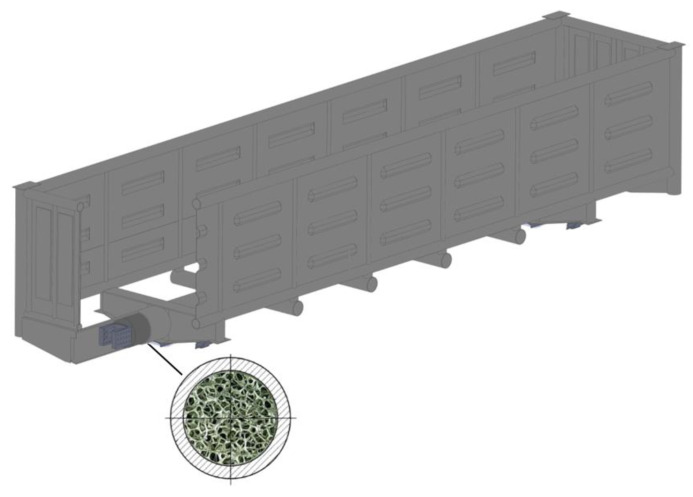
Carrying structure of the circular tube open wagon with aluminum foam as filler for center sill.

The dynamic loading on a wagon in operation can be reduced with the application of aluminum foam as an energy-absorbing material [6,7]. One of the most rational options for using aluminum foam for the wagon structure is to apply it as a filler for wagon components of closed sections, e.g., the circular tube carrying structures of wagons. The authors studied the efficiency in applying aluminum foam in the center sill of an open wagon, as the most loaded unit in the carrying structure (Figure 1).

**Figure 2:**
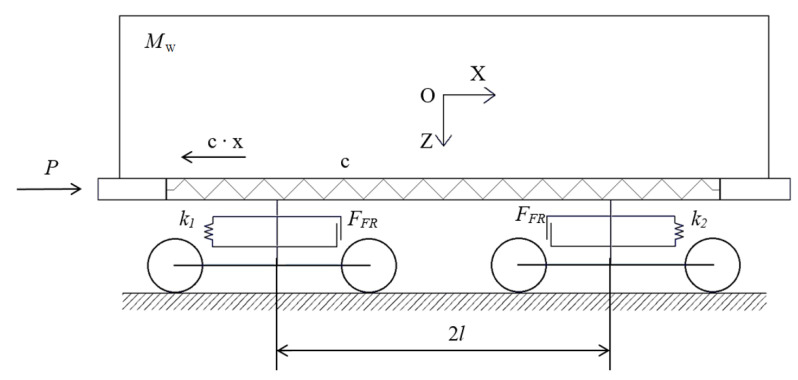
Design diagram of open wagon under shunting impacts.

The dynamic loads on the open wagon body under shunting impacts, as the largest loading on the carrying structure in operation, were defined by the mathematical model developed by Prof. G.I. Bogomaz. The model was composed for the determination of the accelerations as a component of the dynamic loads on a flat wagon loaded with tank containers under shunting impacts. Therefore, it was elaborated for the determination of the accelerations as a component of the dynamic loading on a wagon under the longitudinal impact force. The design diagram is presented in Figure 2. The aluminum foam was considered as an elastic body with a stiffness of 100 kN/m.

(1) $${M^{\prime }}_{W}\cdot {\ddot{x}}_{W}+M^{\prime }\cdot {\ddot{\varphi }}_{W}=P-c\cdot {\dot{x}}_{W};$$

(2) $$I_{W}\cdot {\ddot{\varphi }}_{W}+M^{\prime }\cdot {\ddot{x}}_{W}-g\cdot \varphi_{W}\cdot M^{\prime }=l\cdot F_{FR}\left(sign{\dot{\Delta }}_{1}-sign{\dot{\Delta }}_{2}\right)+l\left(C_{1}-C_{2}\right);$$

(3) $$M_{W}\cdot {\ddot{z}}_{W}=C_{1}+C_{2}-F_{FR}\left(sign{\dot{\Delta }}_{1}-sign{\dot{\Delta }}_{2}\right);$$

where

(4) $${M^{\prime }}_{W}=M_{W}+2\cdot m_{b}+\frac{n\cdot I_{WS}}{r^{2}};\;\;\;M^{\prime }=M_{W}\cdot h;\;\;\;C_{1}=k_{1}\cdot \Delta_{1};\;\;\;C_{2}=k_{2}\cdot \Delta_{2};$$

(5) $$\Delta_{1}=z_{W}-l\cdot \varphi_{W};\;\;\;\Delta_{2}=z_{W}+l\cdot \varphi_{W}.$$

$M_{W}$—mass of carrying structure of the wagon; $I_{W}$—inertia moment of wagon relative to the longitudinal axle; *p*—value of longitudinal impact force to automatic coupler; c—stiffness of the aluminum foam, $m_{b}$—bogie mass; $I_{WS}$—inertia moment of a wheelset; r—radius of the mean worn-out wheel; n—number of bogie’s axles; l—half-base of wagon; $F_{FR}$—absolute value of dry friction force in spring group; $k_{1},\;\;k_{2}$—rigidities of springs in bogie’s suspension; $x_{W},\;\;\varphi_{W},\;\;z_{W}$—coordinates corresponding to the longitudinal, angular around the longitudinal, and the vertical displacements of the wagon, respectively.

**Figure 3:**
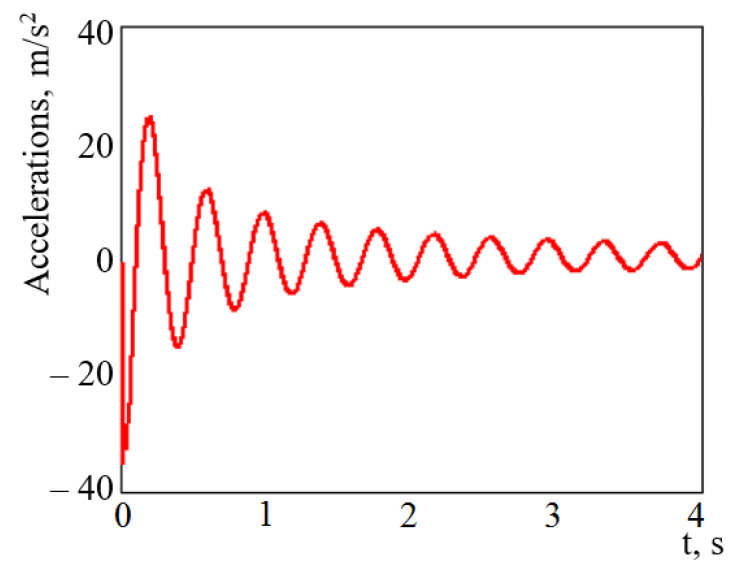
Accelerations on carrying structure of the open wagon under shunting impacts.

The input parameters of the model are the technical characteristics of a wagon, and the value of longitudinal impact force to the automatic coupler. The calculation was made in the case when the longitudinal loading on the carrying structure of an open wagon was P = 3.5 MN [26,28]. The mathematical model was solved in MathCad software (version 15.1, PTC, Boston, MA, USA) [29,30,31]. The initial displacements and the speeds were taken to be equal to zero. The results of the calculation are given in Figure 3.

The maximum accelerations on the carrying structure of an open wagon were 35.7 m/s^2^, which was 3.5% lower in comparison with those for the tube structure without filler.

The finite element method was used for the determination of the strength of an open wagon under shunting impacts [32,33]. The center sill was calculated with a cylindrical element installed inside, with the properties being similar to those of aluminum foam: elastic modulus—5.3 × 10^3^ MPa, Poisson’s ratio—0.3; mass density—800 kg/m^3^; ultimate tensile strength—5.0∙MPa; yield stress—1.05∙MPa.

The optimal number of elements in a grid was determined with the graph-analytical method. Spatial isoparametric tetrahedrons were used as the finite elements; the optimal number of elements was defined with the graph-analytical method. The number of units in the grid was 224,220, and the number of elements was 723,496. The maximum size of an element equaled 70 mm, the minimum size was 14 mm. The minimal number of elements in a circle was 14; the size gain ratio of elements in the grid was 1.8. The maximum side ratio was 707.55, the percentage of elements with a side ratio lower than 3 was 33.2, while percentage of elements with a side ratio more than 10 was 12.8.

**Figure 4:**
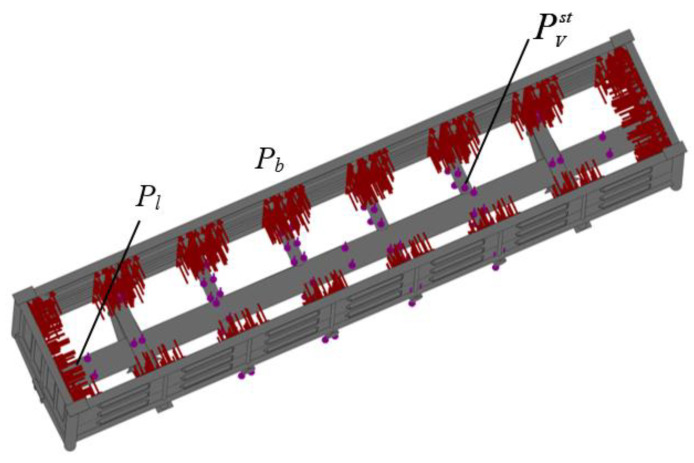
Design diagram of the open wagon.

The design diagram considered that the carrying structure of an open wagon was under vertical static loading $P_{V}^{st}$, longitudinal force $P_{l}$ on the rear support of an automatic coupler, and also under pressure from the bulk freight $P_{b}$ on the side and end walls (Figure 4). The lateral pressure from the bulk freight was calculated by the method given in [34]. Mineral carbon was taken as bulk freight.

**Figure 5:**
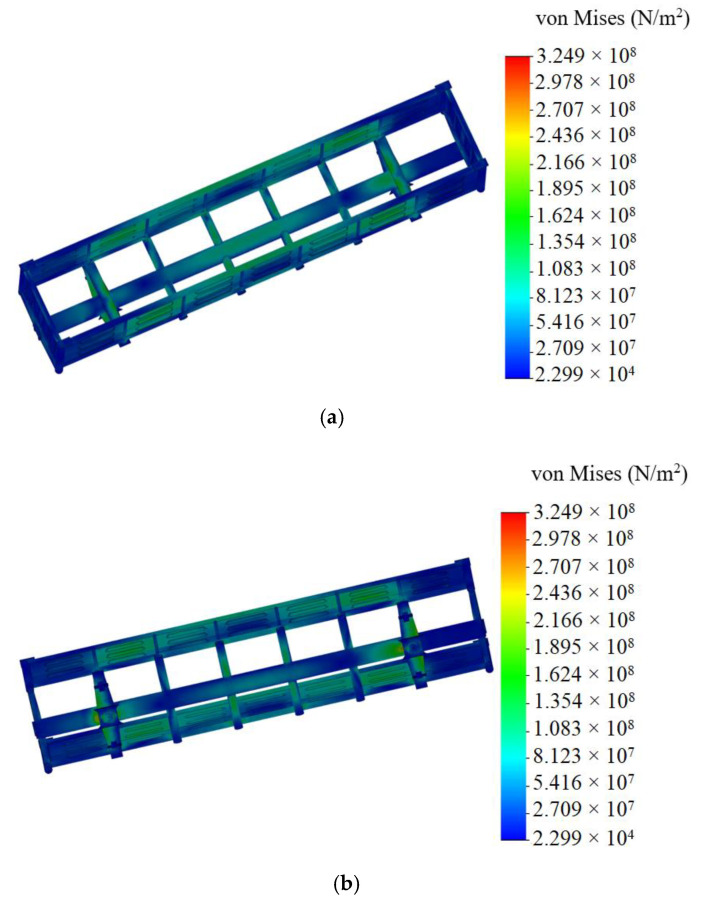
Stressed state of carrying structure of open wagon (**a**) top view; (**b**) bottom view.

Steel 09G2S was taken as the material for the carrying structure of a wagon with the value of the tensile strength σ = 490 MPa and yield stress σ_y_ = 345 MPa. The model was fixed in the areas resting upon the bogies. The results of the calculation are given in Figure 5. The research was carried out for the elastic stage.

**Figure 6:**
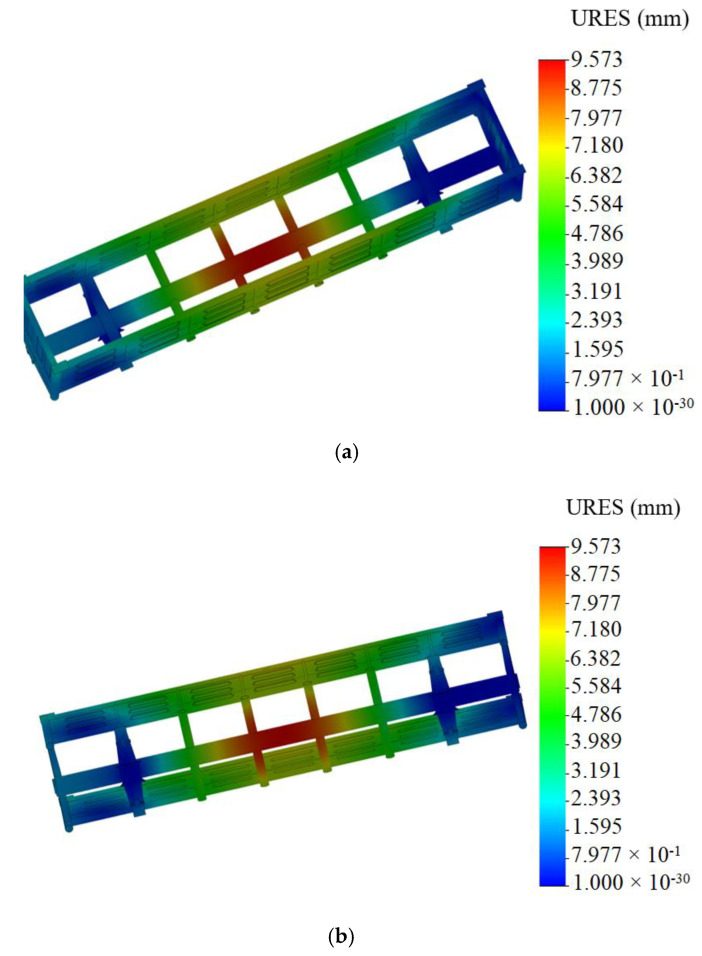
Displacements in assembly units of carrying structure of open wagon (**a**) top view; (**b**) bottom view.

**Figure 7:**
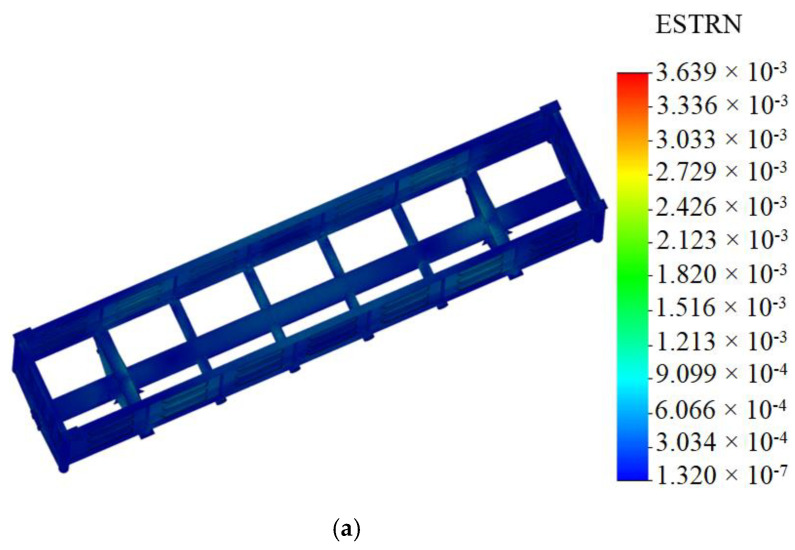

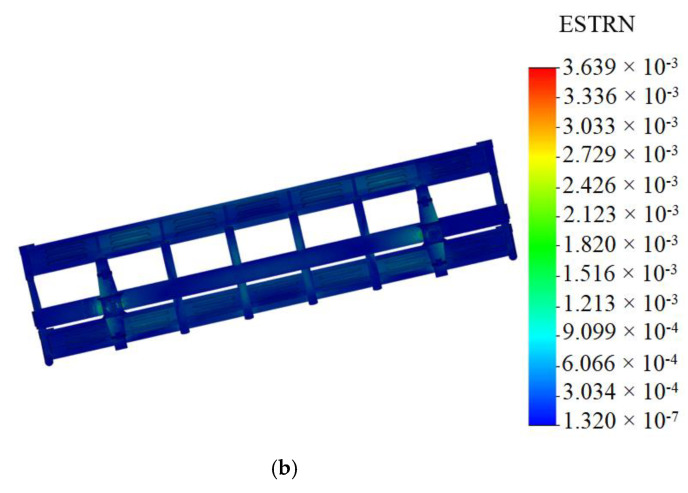
Deformations in carrying structure of open wagon (**a**) top view; (**b**) bottom view.

The maximum equivalent stresses emerged in the bottom part of the center sill behind the rear draft lug and accounted for about 320 MPa. The maximum displacements were fixed in the middle part of the center sill and were 9.5 mm (Figure 6). The maximum deformations were 3.64 × 10^−3^ (Figure 7).

**Figure 8:**
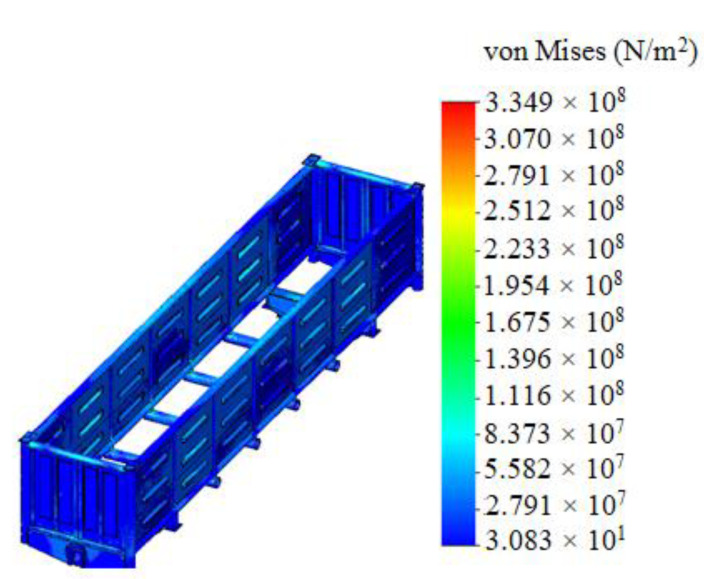
Stress state of the carrying structure of an open wagon without filler.

It was established that foam aluminum applied as the filler for the center sill decreased the maximum equivalent stresses in the carrying structure of an open wagon by about 5% and displacements by 12% in comparison with those of the circular tube carrying structure of an open wagon without the filler (Figure 8). 

The mass of the carrying structure of an open wagon increased by 2.6% in comparison with that of the filler-free structure (at aluminum foam density 300 kg/m^3^).

**Figure 9:**
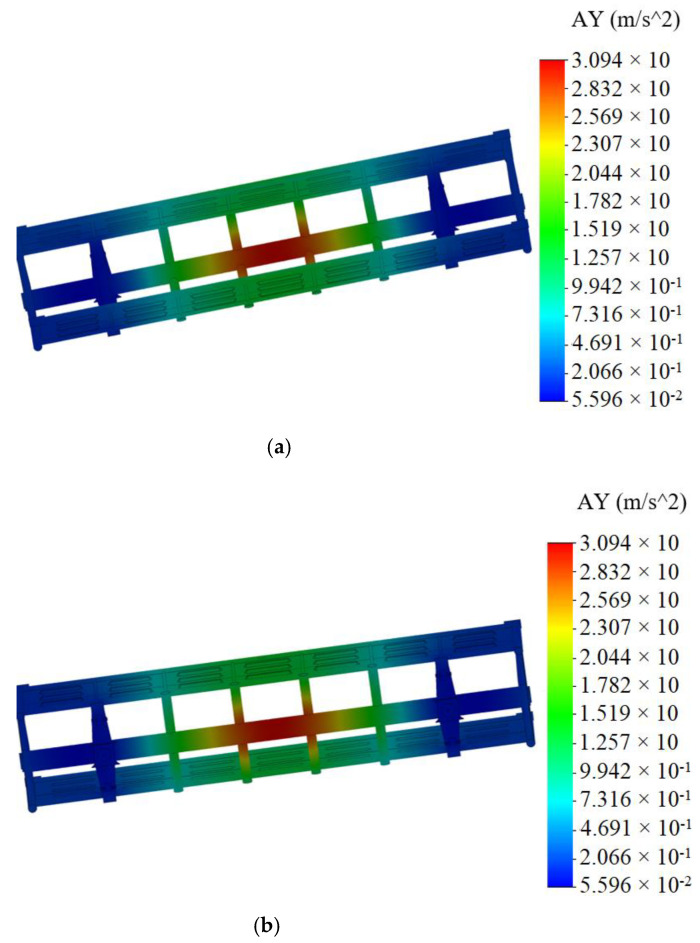
Acceleration distribution fields relative to carrying structure of circular tube open wagon (**a**) top view; (**b**) bottom view.

The acceleration distribution fields relative to the carrying structure of an open wagon for the designed diagram (Figure 4) were determined by computer modeling. Aluminum foam was modeled as an elastic element of the stiffness 100 kN/m. This stiffness value is taken into consideration, since it is with this value that the effectiveness of the proposed solution is traced. The results of the calculation are given in Figure 9.

The maximum accelerations emerging in the mid-span of the center sill were 30 m/s^2^. The maximum accelerations in the side walls were about 20 m/s^2^; they were concentrated in their middle sections. The least acceleration value was in the end parts of the carrying structure of an open wagon.

The natural oscillation frequencies of the carrying structure of an open wagon were determined by a modal analysis in CosmosWorks software [35,36]. The results of the calculation are given in Table 1.

**Figure 10:**
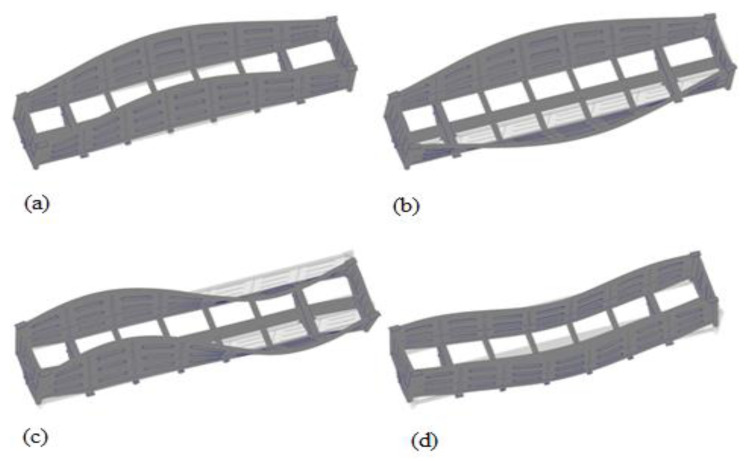
Oscillation modes in carrying structure of open wagon (**a**) 1st natural frequency; (**b**) 2nd natural frequency; (**c**) 3rd natural frequency; (**d**) 4th natural frequency.

Some oscillation modes in the carrying structure of an open wagon are given in Figure 10.

The calculation conducted demonstrates that the natural frequencies are within the admissible values [26,28,37], thus they exceed 8 Hz. In this case, the discrepancy between the first natural vibration frequency of the carrying structure of the open wagon, obtained by mathematical modeling and computer simulation, is about 3%.

**Figure 11:**
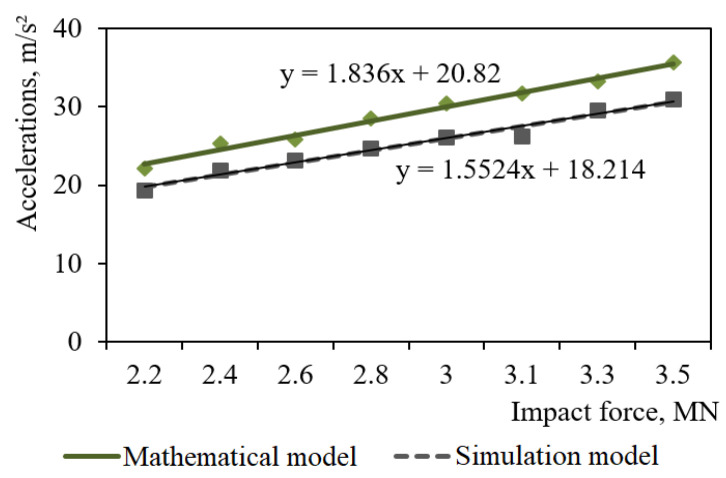
Accelerations on carrying structure of the open wagon at shunting impacts.

The designed dynamic models were checked for adequacy with an F-test [38,39,40]. The input parameter of the model was the impact force to the automatic coupler, and the output was the accelerations on the carrying structure of an open wagon. The results of the calculation are given in Figure 11.

The dispersion of adequacy was $S_{ad}^{2}=20.5$ and the error mean square $S_{S}^{2}=14.8$. Thus, the design value of the criterion was *F_p_* = 1.38, which was lower than its tabular value (*F_t_* = 3.07). It implies that the hypothesis on adequacy is not rejected.

## 5. Conclusions

1. Mathematical modeling of dynamic loads on the carrying structure of an open wagon was conducted with consideration of aluminum foam as the filler for the center sill. The aluminum foam was considered as an elastic body of the stiffness of 100 kN/m. The calculation was made in the case when the longitudinal loading on the carrying structure of an open wagon was P = 3.5 MN. The mathematical model was solved in MathCad software. The maximum accelerations on the carrying structure of an open wagon were 35.7 m/s^2^, which was 3.5% lower in comparison with those of the tubular structure without the filler.
2. The strength of the carrying structure of an open wagon with consideration of aluminum foam as the filler for the center sill was defined by the finite element method in CosmosWorks software with SolidWorks software for the graphics. The maximum equivalent stresses emerging in the top section of the center sill behind the rear draft lugs accounted for about 320 MPa. The maximum displacements fixed in the middle part of the center sill were 9.5 mm. The maximum deformations were 3.64 × 10^−3^. It is established that foam aluminum applied as the filler for the center sill decreases the maximum equivalent stresses in the carrying structure of an open wagon by about 5%, and displacements by 12% in comparison with those of the circular tube carrying structure of an open wagon without filler. The mass of the carrying structure of an open wagon increases by 2.6% in comparison with that of the filler-free structure (at aluminum foam density 300 kg/m^3^).
3. Computer modeling of the dynamic loads on the carrying structure of an open wagon was conducted with consideration of aluminum foam as filler for the center sill. The maximum accelerations emerging in the middle section of the center sill were 30 m/s^2^. The maximum accelerations in the side walls were concentrated in the middle sections and accounted for about 20 m/s^2^. The minimum accelerations were in the end parts of the carrying structure of an open wagon.
4. Modal analysis of the carrying structure of an open wagon with consideration of aluminum foam as filler for the center sill was conducted. It determined the natural frequencies in the carrying structure of a flat wagon. It was established that the values of the natural oscillation frequencies do not fall outside the range of the admissible values.
5. Mathematical models of the dynamic loading of the carrying structure of an open wagon with consideration of aluminum foam as filler for the center sill were verified. A F-test was used as a design criterion. Thus, the adequacy dispersion was $S_{ad}^{2}=20.5$ and the error mean square was $S_{S}^{2}=14.8$. Also, the design criterion value was *F_p_* = 1.38, which was lower than its tabular value (*F_t_* = 3.07). As a result, the hypothesis on adequacy was not rejected.

The research will encourage engineers to design innovative rolling stock units and to improve the operational efficiency.

## Figures and Tables

**Table 1:** Natural oscillation frequencies in carrying structure of the open wagon.

Oscillation Mode	Frequency, Hz	Period, s
1	18.6	0.051
2	19.5	0.041
3	30.9	0.038
4	32.3	0.034
5	33.9	0.033
6	40.3	0.032
7	44.0	0.031
8	48.3	0.030
9	53.3	0.027
10	55.2	0.025

## Data Availability

Not applicable.

## References

[1] Sága M., Blatnický M., Vaško M., Dižo J., Kopas P., Gerlici J. (2020). Experimental Determination of the Manson−Coffin Curves for an Original Unconventional Vehicle Frame. Materials.

[2] Sivanur K., Umananda K.V., Pai D. Advanced materials used in automotive industry-a review. Proceedings of the 3rd International Conference on “Advancements in Aeromechanical Materials for Manufacturing”, ICAAMM 2020.

[3] Lee H.A., Jung S.B., Jang H.H., Shin D.H., Lee J.U., Kim K.W., Park G.J. (2012). A design process for a railway-car body with aluminum extrusion panels using structural optimization. Civ. Comp. Proc..

[4] Hirsch J. (2011). Aluminum in innovative light-weight car design. Mater. Trans..

[5] Findik F., Turan K. (2012). Materials selection for lighter wagon design with a weighted property index method. Mater. Des..

[6] Campana F., Mancini E., Pilone D., Sasso M. (2021). Failure Mechanisms of an Al 6061 Alloy Foam under Dynamic Conditions. Materials.

[7] Costanza G., Tata M.E. (2020). Mechanical behavior of PCMT and SDP Al foams: A comparison. Procedia Struct. Integr..

[8] Dižo J., Blatnický M., Harušinec J., Falendysh A. (2019). Modification and analyses of structural properties of a goods wagon bo-gie frame. Diagnostyka.

[9] Dižo J., Harušinec J., Blatnický M. (2018). Computation of Modal Properties of Two Types of Freight Wagon Bogie Frames Using the Finite Element Method. Manuf. Technol..

[10] Dizo J., Blatnicky M., Skocilasova B. (2015). Computational modelling of the rail vehicle multibody system including flexible bodies. Commun. Sci. Lett. Univ. Zilina.

[11] Stastniak P., Suchánek A., Kurčík P., Smetanka L., Moravčík M. Driveability prediction in design process of freight wag-on underframe. Proceedings of the 17th International Scientific Conference on Dynamics of Rigid and Deformable Bodies 2019.

[12] Štastniak P., Kurčík P., Pavlík A. Design of a new railway wagon for intermodal transport with the adaptable loading platform. Proceedings of the 10th International Scientific Conference Horizons of Railway Transport, HORT 2018.

[13] Šťastniak P., Smetanka L., Moravčík M. (2018). Structural Analysis of a Main Construction Assemblies of the New Wagon Prototype Type Zans. Manuf. Technol..

[14] Antipin D., Racin D., Shorokhov S. (2016). Justification of a Rational Design of the Pivot Center of the Open-top Wagon Frame by means of Computer Simulation. Procedia Eng..

[15] Shukla C.P., Bharti P.K. (2015). Study and Analysis of Doors of BCNHL Wagons. Int. J. Eng. Res..

[16] Harak S.S., Sharma S.C., Harsha S.P. (2014). Structural Dynamic Analysis of Freight Railway Wagon Using Finite Element Method. Procedia Mater. Sci..

[17] Fomin O., Lovska A., Radkevych V., Horban A., Skliarenko I., Gurenkova O. (2019). The dynamic loading analysis of containers placed on a flat wagon during shunting collisions. ARPN J. Eng. Appl. Sci..

[18] Fomin O., Lovska A. (2020). Improvements in passenger car body for higher stability of train ferry. Eng. Sci. Technol. Int. J..

[19] Vatulia G., Komagorova S., Pavliuchenkov M. Optimization of the truss beam. Verification of the calculation results. Proceedings of the 7th International Scientific Conference “Reliability and Durability of Railway Transport Engineering Structures and Buildings” (Transbud-2018).

[20] Bain D.G. (2011). Analysis of stress condition of the runner of floor of the eight-wheel gondola car. J. Vestn. BSTU.

[21] Lee H., Jung S., Jang H., Shin D., Lee J.U., Kim K.W., Park G. (2016). Structural-optimization-based design process for the body of a railway vehicle made from extruded aluminum panels. J. Rail Rapid Transit.

[22] Jang H.J., Shin K.B., Han S.H. (2012). A study on the lightweight design of hybrid modular caibody structures made of sandwich composites and aluminum extrusions using optimum analysis method. Trans. Korean Soc. Mech. Eng..

[23] Fuganti A., Lorenzi L., Grønsund A., Langseth M. (2000). Aluminum foam for automotive applications. Adv. Eng. Mater..

[24] Konyukhov A.D., Zhuravleva L.V., Shurtakov A.K. (2007). Extruded aluminum panels—Perspective material for wagon bodies. Wagons Carriage Facil..

[25] Rahimov R.V., Ruzmetov Y.O. (2018). Analysis of the state and prospects of the development of the freight wagon fleet of the Republic of Uzbekistan. Non-Ferr. Met..

[26] DSTU 7598:2014 (2015). Freight Wagons. General Requirements for Calculations and Design of New and Modernized Wagons of 1520 mm Track (Non-Self-Propelled).

[27] Papantoniou I., Pantelis D., Manolakos D. (2018). Powder metallurgy route aluminium foams: A study of the effect of powder morphology, compaction pressure and foaming temperature on the porous structure. Procedia Struct. Integr..

[28] GOST 33211-2014 (2016). Freight Wagons. Requirements for Strength and Dynamic Properties.

[29] Fomin O., Lovska A. (2020). Establishing patterns in determining the dynamics and strength of a covered freight car, which exhausted its resource. East. Eur. J. Enterp. Technol..

[30] Fomin O., Kulbovsky I., Sorochinska E., Sapronova S., Bambura O. (2017). Experimental confirmation of the theory of implementation of the coupled design of center girder of the hopper wagons for iron ore pellets. East. Eur. J. Enterp. Technol..

[31] Plakhtii O., Tsybulnyk V., Nerubatskyi V., Mittsel N. (2019). The analysis of modulation algorithms and electromagnetic processes in a five-level voltage source inverter with clamping diodes. IEEE International Conference on Modern Electrical and Energy Systems (MEES).

[32] Alyamovsky A.A. (2007). SolidWorks/COSMOSWorks 2006–2007. Finite Element Analysis.

[33] Alyamovsky A.A. (2010). COSMOS Works. Fundamentals of Structural Strength Analysis in SolidWorks.

[34] Lukin V.V., Shadur L.A., Koturanov V.I., Khokhlov A.A., Anisimov P.S. (2000). Design and Calculation of Wagons.

[35] Kondratiev A., Gaidachuk V., Nabokina T., Tsaritsynskyi A. (2020). New possibilities in creating of effective composite size-stable honeycomb structures designed for space purposes. Adv. Intell. Syst. Comput..

[36] Lovska A., Fomin O. (2020). A new fastener to ensure the reliability of a passenger coach car body on a railway ferry. Acta Poly-Tech..

[37] EN 12663–2 (2010). Railway Applications—Structural Requirements of Railway Vehicle Bodies—Part 2: Freight Wagons. B.

[38] Fomin O. (2014). Modern requirements to carrying systems of railway general-purpose gondola cars. Sci. Tech. J..

[39] Lovska A.O. (2015). Computer simulation of wagon body bearing structure dynamics during transportation by train ferry. East. Eur. J. Enterp. Technol..

[40] Plakhtii O., Nerubatskyi V., Sushko D., Ryshchenko I., Tsybulnyk V., Hordiienko D. (2019). Improving energy characteristics of ac electric rolling stock by using the three-level active four-quadrant rectifiers. East. Eur. J. Enterp. Technol..

